# True-Positive ^18^F-Flotufolastat Lesions in Patients with Prostate Cancer Recurrence with Baseline-Negative Conventional Imaging: Results from the Prospective, Phase 3, Multicenter SPOTLIGHT Study

**DOI:** 10.2967/jnumed.123.267271

**Published:** 2024-07

**Authors:** Mark T. Fleming, Rick Hermsen, Andrei S. Purysko, Albert Chau, Phillip Davis, Brian F. Chapin, David M. Schuster, SPOTLIGHT Study Group, Mohamad Allaf

**Affiliations:** 1 Virginia Oncology Associates, US Oncology Network, Norfolk, Virginia;; 2 Department of Nuclear Medicine, Canisius Wilhelmina Ziekenhuis, Nijmegen, The Netherlands;; 3 Section of Abdominal Imaging and Nuclear Radiology Department, Cleveland Clinic, Cleveland, Ohio;; 4 Blue Earth Diagnostics Ltd., Oxford, United Kingdom;; 5 Blue Earth Diagnostics Inc., Monroe Township, New Jersey;; 6 Department of Urology, University of Texas MD Anderson Cancer Center, Houston, Texas; and; 7 Division of Nuclear Medicine and Molecular Imaging, Department of Radiology and Imaging Sciences, Emory University, Atlanta, Georgia

**Keywords:** ^18^F-flotufolastat, PSMA, rhPSMA, prostate cancer, biochemical recurrence

## Abstract

^18^F-rhPSMA-7.3 (^18^F-flotufolastat) is a high-affinity prostate-specific membrane antigen–targeted diagnostic radiopharmaceutical for PET imaging in patients with prostate cancer. Here, we report findings from the SPOTLIGHT study (NCT04186845), assessing the performance of ^18^F-flotufolastat PET/CT for identifying prostate-specific membrane antigen–positive lesions confirmed by standard of truth (SoT) in men with biochemical recurrence of prostate cancer and negative conventional imaging at baseline. **Methods:** Men with biochemical recurrence received 296 MBq of ^18^F-flotufolastat intravenously and then underwent PET/CT 50–70 min later. ^18^F-flotufolastat PET/CT findings were evaluated by 3 masked central readers and verified using histopathology or follow-up confirmatory imaging (CT, MRI, bone scan, or ^18^F-fluciclovine PET/CT) as the SoT. The present analysis evaluated all patients who had negative conventional imaging at baseline, underwent ^18^F-flotufolastat PET/CT, and had SoT verification by histopathology or follow-up confirmatory imaging to report detection rate (DR), which is the number of patients with at least 1 PET-positive lesion, divided by the number of evaluable patients, and verified DR (VDR), which is the proportion of patients with at least 1 true-positive lesion as verified by SoT, of all patients scanned (PET-positive and PET-negative scans). DR and VDR were calculated and stratified according to prior therapy. Majority read data (agreement between ≥2 readers) are reported. **Results:** In total, 171 patients with negative baseline conventional imaging and SoT by histopathology or post-PET confirmatory imaging were evaluated. By majority read, the overall ^18^F-flotufolastat DR among these patients was 95% (163/171; 95% CI, 91.0%–98.0%), and 110 of 171 of these patients had at least 1 true-positive lesion identified (VDR, 64%; 95% CI, 56.7%–71.5%). In the postprostatectomy group (133/171), 8.3% of patients had at least 1 true-positive lesion in the prostate bed, 28% in pelvic lymph nodes, and 35% in other sites. Among those who had received radiotherapy (36/171), 50% of patients had true-positive detections in the prostate, 8.3% in pelvic lymph nodes, and 36% in other sites. **Conclusion:** ^18^F-flotufolastat frequently identified true-positive prostate cancer lesions in patients with negative conventional imaging. ^18^F-flotufolastat may help to better define sites of disease recurrence and inform salvage therapy decisions than does conventional imaging, potentially leading to improved outcomes.

## Visual Abstract

**Figure:**
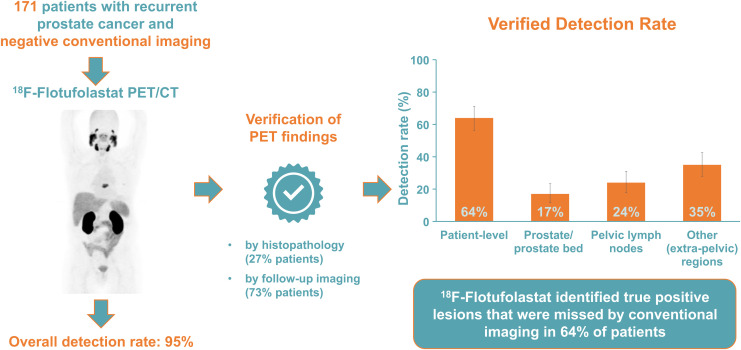

To effectively manage recurrent prostate cancer, it is essential to have accurate imaging techniques since the presence, volume, and distribution of disease will determine therapeutic choices (1). Prostate-specific membrane antigen (PSMA)–targeting radiopharmaceuticals have recently been included in the National Comprehensive Cancer Network, the American Society of Clinical Oncology guidelines, and the European guidelines for prostate cancer detection and selection for radiopharmaceutical therapy with PSMA-targeted agents, such as ^177^Lu-PSMA-617 (2–4). PSMA-targeted PET radiopharmaceuticals such as ^18^F-DCFPyL (^18^F-piflufolastat; Pylarify; Lantheus) and ^68^Ga-PSMA-11 offer improved sensitivity, specificity, and overall diagnostic accuracy compared with conventional imaging methods such as CT, MRI, and bone scintigraphy (2*,*^4^).

The PSMA-targeting radiopharmaceutical ^18^F-flotufolastat (^18^F-rhPSMA-7.3) has recently been approved by the U.S. Food and Drug Administration for diagnostic PET imaging of patients with unfavorable intermediate- and high-risk prostate cancer who are candidates for initial definitive therapy and for patients with suspected biochemical recurrence (BCR) based on elevated serum prostate-specific antigen (PSA) levels (5). ^18^F-flotufolastat is a high-affinity PSMA-targeting PET radiopharmaceutical that is representative of a novel class of radiohybrid PSMA ligands that can be labeled with ^18^F for diagnostic imaging or with α- or β-emitting radiometals for systemic radiation therapy (6). ^18^F-flotufolastat is a single diastereoisomer of ^18^F-rhPSMA-7 that shows high PSMA binding affinity and internalization by PSMA-expressing cells and a favorable diagnostic performance in patients with prostate cancer (6–9). ^18^F-flotufolastat was selected for clinical development on the basis of preclinical assessments (7). Its favorable biodistribution profile in healthy volunteers and patients with prostate cancer (10–12) correlates with clinical data that show the low average urinary excretion of ^18^F-flotufolastat does not impact image assessment for most patients (13). Primary data from the phase 3 SPOTLIGHT study (NCT04186845) show high detection rates (DRs) and standard-of-truth (SoT)–verified DRs (VDRs) with ^18^F-flotufolastat for the accurate localization of recurrent prostate cancer across wide-ranging PSA values, alongside a favorable safety profile (14–16).

In this predefined exploratory analysis of the SPOTLIGHT study, we investigate the added clinical value of ^18^F-flotufolastat PET/CT over conventional imaging and assess ^18^F-flotufolastat PET/CT findings of local, nodal, and metastatic disease in patients with BCR and negative conventional imaging and patients who had SoT verification of PET/CT findings by histopathology or follow-up confirmatory imaging.

## MATERIALS AND METHODS

### Study Design and Patients

SPOTLIGHT was a phase 3, prospective, multicenter, open-label, single-arm study (15). As previously reported, the study protocol was approved by the independent ethics committee from each study site, and all patients provided written informed consent before enrollment (15). Men older than 18 y with previously treated and localized prostate cancer and a PSA level after radical prostatectomy (RP) (with or without radiotherapy) of at least 0.2 ng/mL (with confirmation), or nadir plus 2 ng/mL after radiotherapy, were eligible for inclusion if they were being considered for curative-intent salvage therapy (15).

### Baseline Assessments

Recent conventional imaging was accepted if it had been collected 90 d or fewer before screening. If no conventional imaging was available, this was performed during the baseline assessments (primarily MRI, CT, bone scintigraphy, and ^18^F-fluciclovine PET/CT) (15).

### Imaging Procedures

^18^F-flotufolastat PET/CT took place on day 1, as previously described (15). All PET/CT images were first read onsite to guide SoT verification activities before being sent for interpretation by 3 masked independent central readers. PET/CT findings were verified using histopathology or follow-up confirmatory imaging (CT, MRI, bone scan, or ^18^F-fluciclovine PET/CT) as the SoT within 90 d after ^18^F-flotufolastat PET/CT. When feasible, image-guided biopsies of suspected lesions identified by ^18^F-flotufolastat PET/CT were performed within 60 d. A SoT consensus panel reviewed all available images using prespecified criteria (15).

### Efficacy Endpoints

The overall patient-level DR was the number of patients with at least 1 PET-positive lesion divided by the number of patients with an evaluable PET scan. The VDR was the proportion of patients with at least 1 true-positive lesion verified by SoT, regardless of any coexisting false-positive lesions, of all patients scanned (including both PET-positive and PET-negative patients) (15). Any ^18^F-flotufolastat PET-positive lesion not proven as a true-positive lesion by the SoT was categorized as a false-positive lesion by default. DR and VDR data were analyzed according to the patients’ prior therapy and are presented at a patient level as well as by region: prostate or prostate bed, pelvic lymph nodes, or other (extrapelvic nodes, bone, viscera, and other soft tissues).

### Statistical Analysis

The efficacy analysis population comprised all patients who underwent ^18^F-flotufolastat imaging and had sufficient data for SoT determination (15). The present analysis focuses on patients in the efficacy analysis population who had negative conventional imaging at baseline and SoT confirmation through histopathology or follow-up confirmatory imaging. Imaging endpoints were summarized as point estimates (percentages) for the majority read (agreement between ≥2 readers), alongside 2-sided 95% CIs.

## RESULTS

### Patients

**FIGURE 1.**
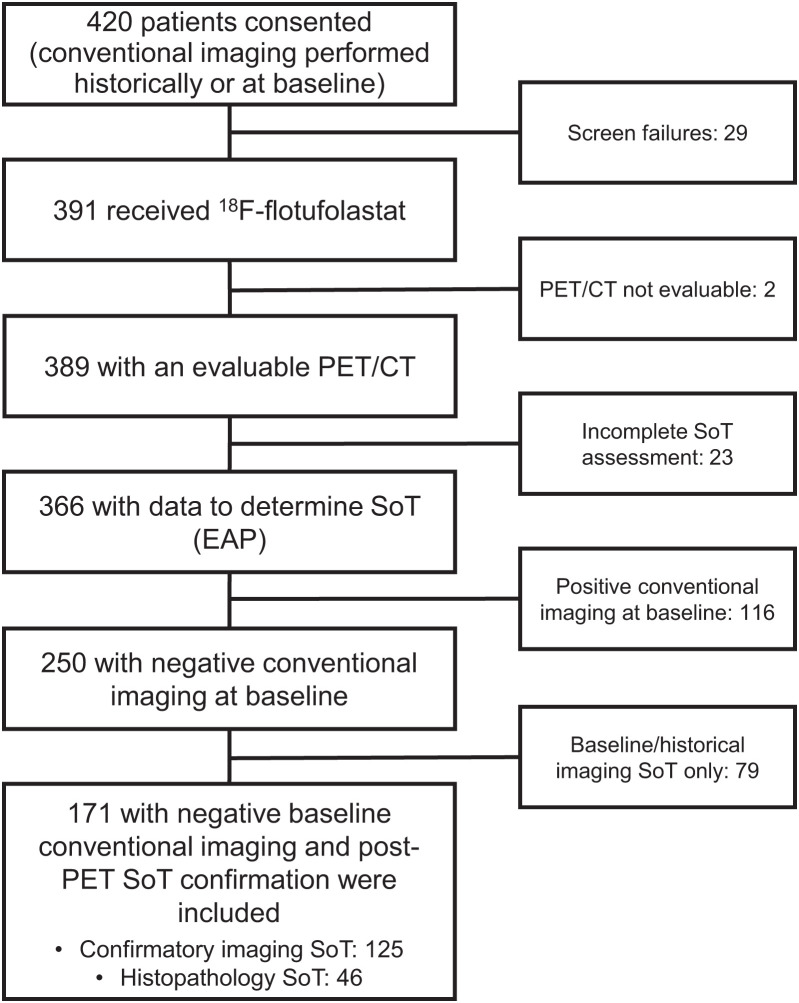
Standards for Reporting of Diagnostic Accuracy flow diagram of study participants. EAP = efficacy analysis population.

In total, 250 of 366 (68%) patients in the efficacy analysis population had negative conventional imaging at baseline. Of these patients, 171 of 250 (68%) had SoT determination based on histopathology (*n* = 46) or follow-up confirmatory imaging (*n* = 125) (Fig. 1). The baseline characteristics for these patients are provided in Table 1.

**TABLE 1.** Patient Baseline Characteristics and ^18^F-Flotufolastat PET/CT and SoT Details

Parameter	Patients with negative baseline conventional imaging and post-PET SoT (*n* = 171)
Age (y)	69 (45–85)
Gleason score	
≤6	12 (7.0%)
7	101 (59%)
≥8	53 (31%)
Missing	5 (2.9%)
ISUP grade group	
1	12 (7.0%)
2	44 (26%)
3	52 (30%)
4	21 (12%)
5	32 (19%)
Missing	10 (5.8%)
Time from initial prostate cancer diagnosis (mo)	71 (2–409)
Prior therapy	
With prior prostatectomy	133 (78%)
With radiotherapy	67 (39%)
Without radiotherapy	66 (39%)
Without prior prostatectomy	38 (22%)
With radiotherapy	36 (21%)
With other therapy	2 (1.2%)
With no prior therapy	0 (0%)
Baseline PSA for all patients (ng/mL)	1.40 (0.20–48.70)
Patients with PSA <1 ng/mL	71 (42%)
Patients with PSA <2 ng/mL	95 (56%)
Baseline PSA for patients treated with prior prostatectomy with or without radiotherapy (ng/mL)*	0.85 (0.20–22.71)
Baseline PSA for patients treated with prior radiotherapy only (ng/mL)^†^	4.08 (1.11–48.70)^‡^
^18^F-Flotufolastat	
Administered activity in MBq	306.3 (230.14–355.20)
Administered activity in mCi	8.28 (6.22–9.60)
SoT modality	
Histopathology	46 (27%)
Imaging only^¶^	125 (73%)
MRI	62 (36%)
CT	35 (20%)
Bone scan	5 (2.9%)
^ 18^F-fluciclovine	37 (22%)

* Two patients received other therapies (*n* =133).

† Two patients received other therapies (*n* = 36).

‡ One patient in SPOTLIGHT efficacy analysis population was found on reevaluation to have prescan PSA value that did not meet inclusion criterion. This patient was excluded from per-protocol population as previously reported (15).

¶ Some patients were evaluated with more than 1 imaging technique.

ISUP = International Society of Urological Pathology.

Qualitative data are number and percentage. Continuous data are median and range.

### ^18^F-Flotufolastat–Positive Lesions in Patients with Negative Conventional Imaging

**FIGURE 2.**
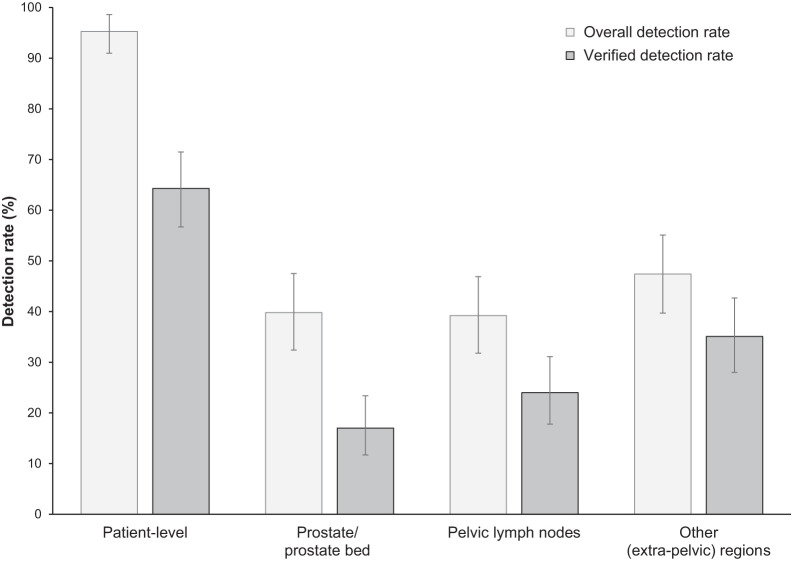
Patient- and region-level overall DRs and VDRs (majority read) for ^18^F-flotufolastat PET/CT in patients with negative conventional imaging and post-PET SoT (*n* = 171).

The overall ^18^F-flotufolastat DR in patients with negative baseline imaging and post-PET SoT confirmation was 95% (163/171; 95% CI, 91.0%–98.0%) by majority read (Fig. 2; Supplemental Fig. 1; supplemental materials are available at http://jnm.snmjournals.org). The VDR was 64% (110/171; 95% CI, 56.7%–71.5%) (Fig. 2).

Regional ^18^F-flotufolastat PET/CT DRs by majority read (Fig. 2; Supplemental Fig. 1) were 40% in the prostate or prostate bed, 39% in pelvic lymph nodes, and 47% in other sites. Verification of these lesions (predominantly by imaging) gave the following VDRs: 17% in the prostate or prostate bed, 24% in pelvic lymph nodes, and 35% in other sites (Fig. 2).

### ^18^F-Flotufolastat PET/CT DR by SoT Modality

**FIGURE 3.**
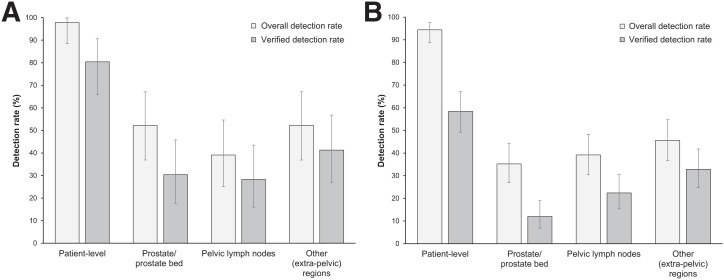
Patient- and region-level overall DRs and VDRs for ^18^F-flotufolastat PET/CT (majority read) in patients with negative baseline conventional imaging and histopathology SoT (*n* = 46) (A) or post-PET imaging SoT (*n* = 125) (B).

In 46 of 171 patients (27%) who had histopathology data available for SoT, the overall DR and VDR were 98% and 80%, respectively. By region, the DR and VDR were 52% and 30% in the prostate or prostate bed, 39% and 28% in pelvic lymph nodes, and 52% and 41% in other sites, respectively (Fig. 3A).

In patients with post-PET confirmatory imaging SoT (125/171, 73%), the overall DR was 94% and the VDR was 58%. Regional DR and VDR were 35% and 12% in the prostate or prostate bed, 39% and 22% in pelvic lymph nodes, and 46% and 33% in other sites, respectively (Fig. 3B).

### ^18^F-Flotufolastat PET/CT DR by Baseline PSA Category

**FIGURE 4.**
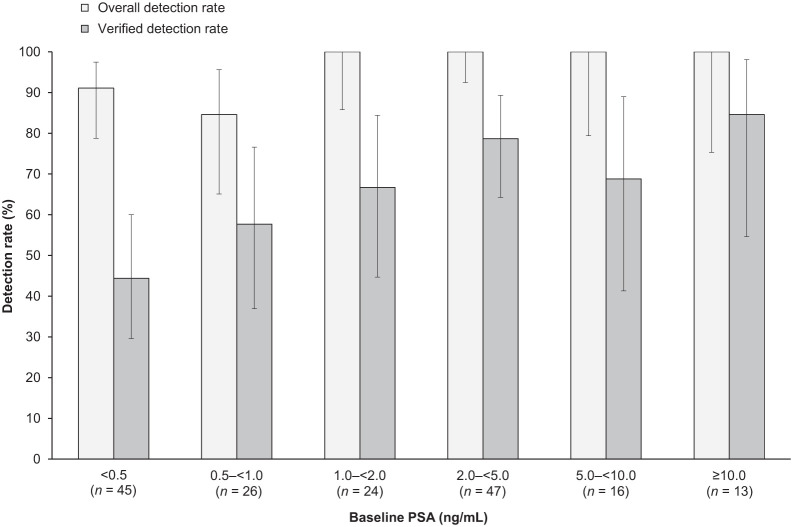
Patient- and region-level overall DRs and VDRs for ^18^F-flotufolastat PET/CT (majority read) by baseline PSA category in patients with negative baseline conventional imaging and post-PET SoT.

Figure 4 shows the ^18^F-flotufolastat PET/CT DR and VDR results by baseline PSA levels. Both DR and VDR broadly increased according to baseline PSA. DR ranged from 91% at a PSA of less than 0.5 ng/mL to 100% at a PSA of at least 1.0 ng/mL. VDR ranged from 44% to 85% across PSA categories, respectively.

### ^18^F-Flotufolastat PET/CT DR by Prior Treatment

**FIGURE 5.**
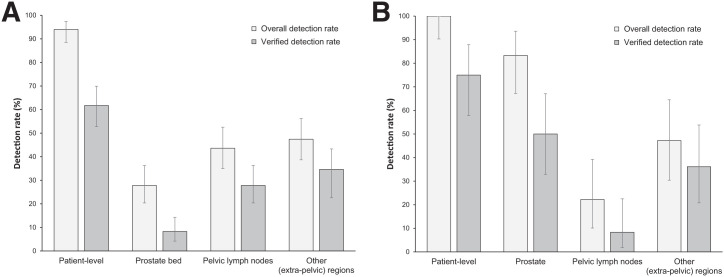
Patient- and region-level overall DRs and VDRs for ^18^F-flotufolastat PET/CT (majority read) in patients with negative baseline conventional imaging and post-PET SoT treated with prior prostatectomy (*n* = 133) (A) or prior radiotherapy only (*n* = 36) (B).

Overall DR and VDR were found to vary according to prior treatment (Figs. 5A and 5B). Among the patients treated with RP (*n* = 133), 33 (25%) had a histopathology SoT available and 100 (75%) had follow-up confirmatory imaging as the SoT. The patient-level DR among the patients with prior RP was 94%. The DR was 28% in the prostate bed, 44% in pelvic lymph nodes, and 47% in other sites (Fig. 5A). The patient-level VDR was 62%, and by region, the VDR was 8.3% in the prostate bed, 28% in pelvic lymph nodes, and 35% in other sites (Fig. 5A).

Among those patients who had received prior radiotherapy only (*n* = 36), 13 (36%) had a histopathology SoT and 23 (64%) had follow-up confirmatory imaging as the SoT. The overall DR for the patients with prior radiotherapy only was 100%. By region, the DR was 83% in the prostate, 22% in pelvic lymph nodes, and 47% in other sites (Fig. 5B). The overall VDR was 75%. The VDR was 50% in the prostate, 8.3% in pelvic lymph nodes, and 36% in other sites (Fig. 5B).

Very few patients had an alternative prior therapy (*n* = 2); therefore, no definitive conclusions could be drawn for them.

**FIGURE 6.**
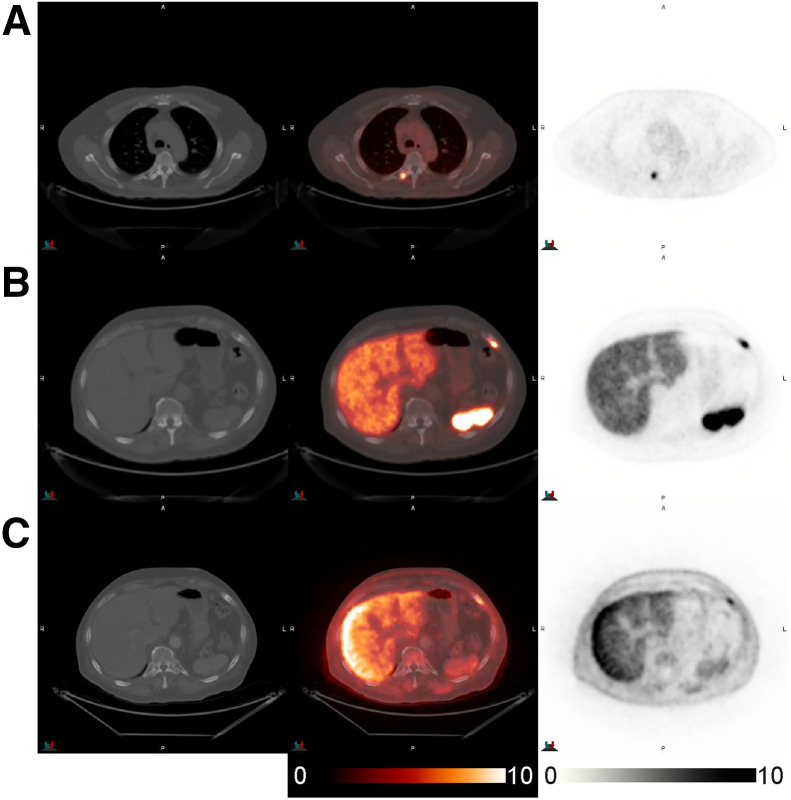
CT (left), fused ^18^F-flotufolastat PET/CT (middle), and ^18^F-flotufolastat PET (right) transverse images of 76-y-old patient initially presenting with high-risk prostate cancer (Gleason score, 4 + 5 = 9) and subsequently with BCR after radical prostatectomy (PSA, 1.0 ng/mL). ^18^F-flotufolastat–avid focus was detected within mildly sclerotic right thoracic 5 transverse process (A) and was subsequently verified as true-positive by histopathology. In addition, ^18^F-flotufolastat–avid lesion was detected in left seventh rib (B) and was also verified with ^18^F-fluciclovine PET/CT (C).

Figure 6 shows ^18^F-flotufolastat PET/CT images from a patient in this cohort with BCR after radiotherapy (PSA, 1 ng/mL).

## DISCUSSION

The prospective, phase 3, multicenter SPOTLIGHT study has reported the diagnostic utility of ^18^F-flotufolastat PET/CT in men with BCR of prostate cancer (15). In this exploratory analysis, we assessed the ability of ^18^F-flotufolastat PET/CT to identify true-positive (SoT-verified) lesions in the subgroup of SPOTLIGHT patients who had negative conventional imaging at baseline and sufficient data for SoT determination by histopathology or follow-up confirmatory imaging. Our data demonstrate a high likelihood of positive results from ^18^F-flotufolastat PET/CT even when conventional imaging is negative.

Conventional imaging has limited utility for localizing recurrent disease, especially at low PSA levels. For instance, in patients with BCR after RP, only 11%–14% of patients have a positive CT scan (4*,*^17^), and previous studies have shown that the mean PSA level associated with a positive CT scan can be as high as 27 ng/mL (17*,*^18^). Similarly, a positive bone scan is more likely at a PSA level of at least 20 ng/mL and unlikely at a PSA level of less than 7 ng/mL (17*,*^19^). PSMA-based PET offers sensitive imaging at low PSA levels (<0.5 ng/mL) and may help distinguish patients with local recurrence or locoregional spread from those with distant disease after RP, which may influence subsequent treatment choices, including salvage radiotherapy (4). In line with this, and because of the higher sensitivity and specificity of PSMA PET for the detection of micrometastatic disease, especially at low PSA levels, the current National Comprehensive Cancer Network and American Society of Clinical Oncology prostate cancer guidelines recommend PSMA PET (such as with ^18^F-flotufolastat, ^18^F-piflufolastat, or ^68^Ga-PSMA-11-PET) as a front-line imaging tool for patients with BCR or during initial staging or as a work-up for progressive disease and patient selection for PSMA radiopharmaceutical therapy (2*,*^3^).

The SPOTLIGHT study reported an overall DR of 83% among 389 patients with BCR (PSA, 1.10 ng/mL; range, 0.03–134.6 ng/mL) (15). In our current analysis of patients with negative baseline imaging and post-PET SoT data (*n* = 171), the median baseline PSA value was 1.40 ng/mL, with 42% of patients having a PSA of less than 1.00 ng/mL. Our overall DR (95%) in these patients compares favorably with data for other PSMA PET radiopharmaceuticals. Data from the CONDOR study show an overall ^18^F-piflufolastat DR of 59%–66% in patients with BCR and negative or equivocal standard-of-care imaging (20), and an overall DR of 75% has been reported for ^68^Ga-PSMA-11 PET in patients with BCR (median PSA, 2.1 ng/mL), although these patients were included regardless of prior conventional imaging findings (21).

The overall DR in patients treated with prior radiotherapy (100%) was higher than that in patients who had prior RP (94%), and a similar pattern was observed in the prostate region. This is likely a reflection of higher baseline PSA levels in postradiotherapy patients (to meet the current criteria for BCR (22)). When DR and VDR for ^18^F-flotufolastat PET/CT were analyzed by PSA category, the data indicated that both DR and VDR broadly increased according to baseline PSA (Fig. 4).

Although region-level DRs were moderate in some regions, likely because not every patient will have recurrent lesions in every region evaluated, the patient-level DR of 95% shows the high likelihood that ^18^F-flotufolastat PET/CT will localize recurrent lesions among patients with suspected recurrence of prostate cancer, even in cases where conventional imaging is negative. Moreover, the use of the robust and new recommended metric by the U.S. Food and Drug Administration for BCR imaging trials, VDR, demonstrates how ^18^F-flotufolastat PET/CT provides clinically meaningful information. The VDR of 64% observed across all patients with negative imaging at baseline indicates that ^18^F-flotufolastat PET/CT enabled visualization of at least 1 true-positive lesion in nearly two thirds of patients that had been missed by conventional imaging. Accurate localization of these recurrent lesions can help inform patient management (23*,*^24^), which may result in improved outcomes (25*,*^26^).

It is worth noting that only follow-up conventional imaging (primarily MRI or bone and CT scans) was available as the SoT for most patients in this analysis (73%), rather than histopathology, which was available in only 27% of cases (at least partly due to the coronavirus disease 2019 pandemic); therefore, the VDR might partly be driven by false false-positive results due to a predominance of conventional imaging–only SoT; that is, a substantial proportion of false-positive lesions may have been proven to be true-positive lesions if histopathology had been available in a higher percentage of patients or if longer-term follow-up of these patients was available. In line with this, we show the VDR to be greater in patients with histopathology SoT than in patients with imaging SoT and closer to the overall DR, indicating that fewer PET-positive lesions are considered to be false-positive lesions when histopathology is used as the SoT. Currently, the National Comprehensive Cancer Network guidelines recommend histologic confirmation of any PET findings wherever possible to rule out false positives (2). Nonetheless, false positives are increasingly outweighed by the improved true-positive DR by PSMA PET imaging compared with conventional imaging (2), which has led to its recommendation over conventional imaging modalities for guiding treatment choices in patients with BCR (2–4).

## CONCLUSION

The present findings from the SPOTLIGHT study show that ^18^F-flotufolastat PET/CT frequently detects true-positive lesions among patients otherwise considered negative for recurrence by conventional imaging (particularly among patients with intact prostates). It may help to inform when to omit potentially toxic local treatment when finding multifocal metastatic disease. Such findings may help to better define sites of disease recurrence and inform salvage therapy decisions than is possible with conventional imaging alone.

## DISCLOSURE

Mark Fleming has received travel expenses from Blue Earth Diagnostics. Rick Hermsen reports consultancy fees from Blue Earth Diagnostics and ABX GmbH. Andrei Purysko holds grants or contracts from the American College of Radiology, Blue Earth Diagnostics, and Koelis and has received consulting fees or honoraria from Blue Earth Diagnostics and Koelis. Albert Chau received consultancy fees from Blue Earth Diagnostics for data management and statistical services. Phillip Davis is an employee of Blue Earth Diagnostics. David Schuster has acted as a consultant for Global Medical Solutions Taiwan, Progenics Pharmaceuticals, Inc., Heidelberg University, and DuChemBio Co. Ltd.; participates through the Emory Office of Sponsored Projects in full compliance with Emory University sponsored research and conflict-of-interest regulations in sponsored grants including those funded or partially funded by Blue Earth Diagnostics, Nihon MediPhysics Co, Ltd., Telix Pharmaceuticals (US) Inc., Advanced Accelerator Applications, FUJIFILM Pharmaceuticals USA, Inc., Amgen Inc.; participates in educational initiatives with School of Breast Oncology and PrecisCa; and provides medicolegal consulting vetted through Emory SOM. This study was funded by Blue Earth Diagnostics Ltd., Oxford, U.K. No other potential conflict of interest relevant to this article was reported.

## Notes

KEY POINTS **QUESTION:** Can ^18^F-flotufolastat PET/CT detect lesions missed by conventional imaging in patients with suspected recurrence of prostate cancer? **PERTINENT FINDINGS:** In patients with negative baseline conventional imaging, the overall ^18^F-flotufolastat PET/CT DR was 95%, and 110 of 171 of these patients had at least 1 true-positive lesion verified by histopathology or follow-up confirmatory imaging (VDR, 64%). Overall DR and VDR varied according to the SoT modality and by prior therapy, with higher VDRs in patients with histopathology and in men treated with prior radiotherapy only. ^18^F-flotufolastat PET/CT showed high levels of detection across a range of PSA values, including in patients with PSA levels less than 0.5 ng/mL. **IMPLICATIONS FOR PATIENT CARE:** As ^18^F-flotufolastat PET/CT frequently identifies true-positive lesions in patients with negative conventional imaging, it is a useful tool to accurately identify sites of disease recurrence and inform subsequent treatment decisions, potentially improving patient outcomes.
